# Effects of Functional Biomaterials on the Attributes of Orally Disintegrating Tablets Loaded with Furosemide Nanoparticles: In Vitro and In Vivo Evaluations

**DOI:** 10.3390/jfb15060161

**Published:** 2024-06-09

**Authors:** Doaa Alshora, Wejdan Alyousef, Mohamed Ibrahim

**Affiliations:** Department of Pharmaceutics, College of Pharmacy, King Saud University, P.O. Box 22452, Riyadh 11459, Saudi Arabia; wejdzan@gmail.com (W.A.); mhamoudah@ksu.edu.sa (M.I.)

**Keywords:** furosemide, nanoparticles, functional biomaterials, response methodology, ODT, diuretic activity

## Abstract

Furosemide (FUR) is a diuretic used to relieve edema, congestive heart failure, cirrhosis, end-stage renal disease, and hypertension. FUR is a class IV according to the Biopharmaceutics Classification System. It is practically insoluble in water. This study aimed to optimize and formulate porous orally disintegrating tablets (ODTs) prepared by sublimation and loaded with FUR nanoparticles prepared by using a planetary ball mill. Different functional biomaterials called stabilizers were used to stabilize the nanoparticle formula. Pluronic F-127 was the optimum stabilizer in terms of particle size (354.07 ± 6.44), zeta potential (−25.3 ± 5.65), and dissolution efficiency (56.34%). The impact of the stabilizer concentration was studied as well, and a concentration of 3% showed the smallest particle size (354.07 ± 6.44), best zeta potential value (−25.3 ± 5.65), and percentage of dissolution rate (56.34%). A FUR-loaded nanoparticle formula was successfully prepared. The nanoparticle formula was stabilized by using 3% pluronic F-127, and 3% was chosen for further study of the incorporation into oral disintegration tablets prepared by the sublimation technique. The impact of the matrix sublimating agent and superdisintegrant on the ODTs’ attributes (in vitro disintegration, wetting time, and in vitro dissolution efficiency) was studied using 3^2^ full factorial designs. *In vivo*, the diuretic activity was tested for the optimized FUR ODTs by calculating the Lipschitz value using rats as an animal model. The stability of the ODTs loaded with FUR nanoparticles was assessed under accelerated conditions for 6 months. Finally, the ODT formula loaded with FUR NPs showed a rapid onset of action that was significantly faster than untreated drugs. Nanonization and ODT formulation enhances the dissolution rate and bioavailability of FUR. Many factors can be controlled to achieve optimization results, including the formulation and process parameters.

## 1. Introduction

Heart failure (HF) is a health problem caused by cardiac function abnormalities [1]. HF can be characterized by high venous pressure with signs of clinical congestion. This clinical congestion includes hepatic enlargement and pulmonary and peripheral edema [2]. The first line of therapy in this case is to use loop diuretics. Furosemide (FUR) is the most prescribed loop diuretic for patients with HF and renal disease, which enhances the excretion of sodium, chloride, and potassium [3]. The regular dose of FUR is 20–40 mg. In the case of HR, this dose could be increased to 500 mg to have a pharmacological effect [4]. The onset of action in patients with HF and renal disease is very critical. The demands of this high dose could be due to FUR’s poor bioavailability, which ranges from 47–70% for normal patients and 43–46% for end-stage renal disease patients, which can also be translated by a reduction in the diuretic effect by 50% [5]. The reason for this could be related to its solubility behavior. Regarding its solubility and permeability classification, it belongs to class IV, meaning it is poorly soluble and permeable. The biological half-life of FUR is 1.5 h, which demands an increase in the administration frequency, especially in patients with HF and renal disease.

Nanotechnology is widely used in the pharmaceutical field. It plays a crucial role in enhancing drug solubility by decreasing the material size to the nanometer scale. Decreasing the particle size to the nanometer scale may promote a drug’s solubility and dissolution according to the Noyes–Whitney theory [6]. Several approaches have been used to reduce the particle size, and the wet milling technique is one of these approaches. The wet milling technique belongs to the top-down approach, in which micronized particles decrease to nanonized particles in size. The milling technique using a planetary ball mill for particle size reduction is based on centrifugal forces that cause constant changes in the direction and frequency, resulting in an efficient and fast grinding process [7]. The planetary ball mill is an efficient approach that has been utilized to enhance the solubility and bioavailability of several drugs such as Rosuvastain calcium [8,9] and Pioglitazone [10].

The selection of nanoparticle stabilizers is a crucial step toward the stabilization of the produced nanosuspension or nanoparticles after freeze-drying. The selection of the nanoparticles’ stabilizers is dependent on several parameters such as the nanonization technique, mechanism of stabilization, nature of the drug, nature of stabilizer, and route of administration of dosage forms based on nanoparticulate delivery systems [11]

Orally disintegrating tablets (ODTs) are solid dosage forms that dissolve rapidly, usually within seconds of being placed on the tongue. The main advantage of ODTs that they provide rapid dissolution and absorption of the drug, leading to a rapid onset of action. Also, they provide a particular benefit for patients with swallowing difficulties, such as pediatric, geriatric, and psychiatric patients [12]. This novel pharmaceutical technology has been widely used recently to enhance the dissolution and bioavailability of several drugs. ODTs can be manufactured by different techniques such as freeze-drying, spray-drying, and sublimation. In the sublimation method, the drug is compressed with highly volatile sublimating agents such as camphor and thymol, which are volatilized on drying, leaving a porous tablet structure [13].

## 2. Experimental Section

### 2.1. Materials

FUR was obtained from Hebei Guanlang Biotechnology Co., Ltd. (Shijiazhuang, China). Hydroxypropyl methylcellulose, (HPMC, Methocel E5 PREM LV) was acquired from DOW (Midland, MI, USA). Polyvinylpyrrolidone (PVP K30, MW 40000) was obtained from BASF (Los Angeles, CA, USA). Polyoxyethylene sorbitan monooleate (Tween 80^®^) was purchased from Merck Company (Muenchen, Germany). Polyoxyethylene polyoxypropylene block copolymer (Poloxamer 407; pluronic F-127) was obtained from C.H. Erbesloh (Krefeld, Germany). Polyethylene glycol 4000 (PEG 4000) was purchased from Koch-Light Laboratories Ltd. (Colnbrook, Bucks, UK). Polyvinyl alcohol-acrylic acid-methyl methacrylate copolymer (Povacoat^®^ F) was obtained as a gift from Daido Chemical Corporation (Osaka, Japan). Microcrystalline cellulose (MCC; Avicel^®^ PH101) was purchased from Serva Feinbiochemica (Heidelberg, Germany). Lactose was obtained from BDH Company (Mumbai, India). Thymol was obtained from BDH Company (Mumbai, India). Crospovidone (CPV) was kindly supplied by Riyadh Pharma (Riyadh, Saudi Arabia). Potassium dihydrogen orthophosphate and sodium hydroxide were obtained from Varda House, Daryaganj (NewDelhi, India).

### 2.2. Methods

#### 2.2.1. Formulation and Optimization of the FUR Nanoparticles

The FUR nanoparticles were prepared by the beads milling technique using a planetary ball mill (Pulverisette 7 Premium). The optimization of the milling procedures was carried out by screening the effects of different stabilizer types and their concentrations on the quality attribute of the prepared nanoparticles. In the first stage, screening of different stabilizer biomaterials (HPMC E5 LV, PVP K30, Povacoat, pluronic F-127, Tween 80, and PEG 6000) was carried out to select the best one. The nanoparticles were prepared by milling the drug with 3% of each aqueous stabilizer solution at 500 rpm using zirconium balls of 0.5 mm in size and weighing nearly 100 g. The milling was carried out over three cycles, and each cycle period was 10 min. The pause time between the cycles was fixed for 5 min. The prepared nanoparticles were subjected to freeze-drying with a vacuum pressure of less than 1 mbar at −60 °C for at least 2 days [10], and the produced solid nanoparticles were stored in a tight container shielded from light at −30 °C for additional characterization. The selection of the optimum nanoparticle formula in this stage was based on the nanoparticle size, zeta potential, and drug dissolution rate.

In the second stage, the effect of different concentrations (1%, 3%, and 5%) of the selected stabilizer on the nanoparticles’ attributes was then studied using the same milling conditions in the previous stage.

#### 2.2.2. Characterization of FUR Nanoparticles

*Particle Size (PS), Polydispersity Index (PDI), and Zeta Potential (ZP)*

The FUR nanoparticle formulations’ particle size, PDI, and zeta potential values were determined using a Malvern Zetasizer system. The particle size for both the nanoparticles and freeze-dried powder was measured after a certain dilution. The experiment was carried out in triplicate.

*Nanoparticle FUR content*

The FUR content in the nanoparticles was measured spectrophotometrically in a triple manner. An aliquot of 10 mg of nanoparticles was dissolved in 50 mL methanol and filtered using a 0.45 μm nylon filter, from which 10 mL was then withdrawn and diluted to 50 mL with phosphate buffer, pH 6.8. The spectrophotometric absorbance was measured at 274 nm against a suitable blank.

*In vitro dissolution*

A USP-II dissolution apparatus was used to study the *in vitro* dissolution profile of the FUR nanoparticles. The study was conducted in 900 mL of phosphate buffer at pH 6.8 that was warm and adjusted at 37 °C with a paddle rotation of 50 rpm. Aliquots of 5 mL samples were withdrawn at preset times of 5, 10, 15, 30, 45, and 60 min using a poroplast-kerze filter and diluted suitably, and the absorbance was measured spectrophotometrically at 274 nm. The % of FUR dissolved from the nanoparticles was calculated. The dissolution efficiency (DE%) was calculated from the area under the dissolution curve plotted versus the time (measured using the trapezoidal rule) and expressed as a percentage as follows [14]:

$$\mathrm{Dissolution}\;\mathrm{Efficiency}\;(D.E.)=\frac{\int_{o}^{t}y\cdot dt}{y_{100}\cdot t}\times 100\%$$

*Physicochemical characterization of optimizing FUR nanoparticles*

*Nanoparticle morphology (SEM)*

The surface characteristics of the FUR nanoparticles were determined by scanning electron microscopy. The samples were coated with a thin layer of gold–palladium under an argon atmosphere using a gold sputter module with a high-vacuum evaporator. The coated samples were then scanned, and photomicrographs were taken [10].

*X-ray powder diffraction (XRPD)*

The crystalline attribute of the FUR nanoparticles as compared to the individualized component and a physical mix with the polymer was determined by using X-ray diffractometry. The X-ray diffraction patterns of the powder samples were obtained using a diffractometer, which was connected to a curved graphite crystal monochromator, an automatic divergence slit, and a PW/1710automatic controller. The scanning run was continuous with 2Ɵ degrees ranging from 3° to 60° [10].

*Fourier transform infrared spectroscopy (FTIR)*

The FTIR spectra for the FUR raw material, FUR nanoparticles, and polymer was determined. The substances were identified based on spectral analysis over a wavenumber range from 4000 to 500 cm^−1^; potassium bromide (spectroscopic grade) was mixed with the samples and compressed into disks using a hydraulic press. The data were analyzed using the Perkin Elmer (Spectrum V5.3.1) program.

*Differential scanning calorimetry (DSC)*

Thermograms of the FUR nanoparticles compared to the untreated drug (FUR) and the polymer (pluronic F-127) were recorded by DSC for thermal analysis using a DS calorimeter. Samples of 3–5 mg were sealed in aluminum pans and heated at a constant rate of 10 °C/min over a temperature range from 30 °C to 350 °C. Thermograms of the samples were then obtained using a software program in a TA50I PC system.

#### 2.2.3. Formulation of ODTs Loaded with FUR Nanoparticles by the Sublimation Method

*Design of ODT formulations containing optimized FUR NP formula*

FUR ODTs containing the optimized FUR nanoparticles equivalent to 20 mg of the drug were prepared by the sublimation technique. Thymol was used as a sublimating volatile agent and CPV as a superdisintegrant, and a mixture of lactose and Avicel^®^ (1:1) was used as a diluent.

Two independent factors were studied for their impacts on the ODTs’ attributes, namely, thymol (A; at levels of 10, 15, and 20% of the tablet weight) and CPV (B; at levels of 2, 6, and 10% of the tablet weight). These independent factors were studied for their effects on the in vitro disintegration (Y1), wetting time (Y2), initial dissolution rate (IDR; Y3), and in vitro dissolution efficiency (DE%; Y4). The two independent variables as well as their levels are shown in Table 1.

*Manufacture of ODTs using the sublimation technique*

ODTs containing FUR nanoparticles were fabricated by direct compression according to the tablet composition in Table 2. An Avicel–lactose mixture (1:1), thymol, and CPV were weighed and mixed for 10 min. Sodium stearyl fumarate (lubricant) was then added, and the powder was mixed for 2 min. The powder blend was then compressed into 200 mg tablets using a 9 mm (mm) rounded flat punch. Tablets were kept at 60 °C for 1 h in an oven to ensure complete sublimation, which was verified by measuring the weight loss [15].

*Evaluation of sublimated ODTs loaded with FUR nanoparticles.*

*Weight variation*

Twenty tablets from each batch were individually weighed (Analytical balance, Shimadzu, EB-3200D, Tyoto, Japan), and the average weight and deviating values were calculated.

*Dosage unit uniformity*

The drug content uniformity was determined for 10 tablets according to the USP 34 guidelines (USP 34). The guidelines state that the acceptance value (AV) should be less than or equal to 15 for 10 units tested and not more than 25 for 30 units tested [16]. The AV was calculated as follows:

General formula: AV = (X − M) + K·S

where AV is the acceptance value, X is the mean value of the drug content, S is the standard deviation, and K is a constant value that was either equal to 2.4 for 10 dosage units or equal to 2 for 30 dosage units.

In the above expression, there are three cases:

If 98.5% ≤ X ≤ 101.5%, M = X and AV = K·S

If X < 98.5%, M = 98.5% and AV = 98.5% − X + K·S

If X > 101.5%, M = 101.5% and AV = X − 101.5% + K·S

The content uniformity of the furosemide ODTs was analyzed using a UV spectrophotometer at a wavelength of 277 nm. Each tablet was placed in a 100 mL volumetric flask and dispersed in 10 mL of methanol and 50 mL of phosphate buffer (pH 5.8) with the aid of sonication for 20 min. The volume was completed with the buffer. The dispersion was then filtered using a 0.45 µm syringe filter. The sample’s absorbance was measured, and the content was computed.

*Tablet thickness*

Tablet thickness was measured on 10 ODTs using a digital micrometer, and the average thickness and standard deviation were determined.

*In vitro disintegration time*

The determination of the in vitro disintegration time of the sublimated ODTs was carried out according to the USP30-NF25 guidelines using a tablet disintegration tester for six tablets. One tablet was allocated to each of the six tubes of the basket containing 1000 mL of phosphate buffer (pH 6.8) and kept at 37 °C ± 1 °C, and the disintegration time and standard deviation were recorded.

*Wetting time*

A piece of tissue paper was placed in a petri dish containing ten milliliters of colored solution. Three tablets were placed on the paper, and the time required for complete tablet wetting was reported. Complete wetting was taken as the time at which the colored water covered the entire tablet [17]. The test results were presented as the mean value of three determinations.

*In vitro dissolution studies*

The in vitro dissolution study of the FUR nanoparticles and sublimated ODTs was performed using a USP-II dissolution apparatus. The dissolution experiment was carried out in 900 mL of 0.1 N HCl as a dissolution medium at 37 °C and 75 rpm. An aliquot of 5 mL was withdrawn at preset times of 5, 10, 15, 30, and 60 min. The absorbance was measured spectrophotometrically at 277 nm against a suitable blank. The dissolution efficiency percentage after 60 min (DE%) was calculated [14,18].

#### 2.2.4. In Vivo Study of Diuretic Activity in Rats

The diuretic activity assessment was carried out as prescribed by Lipschitz [19]. The rats fasted overnight in terms of food, and free access to water was allowed. All animal experiments were performed as per our institutional animal care guidelines. The use of animals and experimental design were approved by the Scientific Research Ethics Committee, King Saud University [KSU-SE-23-19]. Before the administration of the drug, all the rats’ urinary bladders were emptied by gently compressing the pelvic area and using the pulldown of their tails. Three groups of animals (each of six) were involved in this study. The first group of animals (control group) received 1 g/kg of urea followed by 5.0 mL of 0.9% NaCl solution per 100 g body weight by gavage. The second group received untreated FUR (in a dose of 2 mg/kg) suspended in a 0.5% CMC solution. The third group received the crushed optimized FUR ODT formula equivalent to 2 mg/kg FUR suspended in a 0.5% CMC solution. Immediately after administration, the rats were placed in a metabolic cage, and the excreted urine volume was collected and measured at the end of the 1st h, 2nd h, 4th h, 6th h, and, finally, 24th h intervals after dosing. The urinary excretion, diuretic activity, and diuretic index were calculated according to the equations below.

$$\mathrm{Urinary}\;\mathrm{excretion}=\frac{\mathrm{Total}\;\mathrm{urinary}\;\mathrm{output}\;}{\mathrm{Total}\;\mathrm{volume}\;\mathrm{of}\;\mathrm{liquid}\;\mathrm{administered}}\times 100\;\mathrm{Diuretic}\;\mathrm{action}=\frac{\mathrm{Urinary}\;\mathrm{excretion}\;\mathrm{of}\;\mathrm{test}\;\mathrm{group}\;}{\mathrm{Urinary}\;\mathrm{excretion}\;\mathrm{of}\;\mathrm{control}\;\mathrm{group}}\;\mathrm{Diuretic}\;\mathrm{Index}=\frac{\mathrm{Diuretic}\;\mathrm{action}\;\mathrm{of}\;\mathrm{test}\;\mathrm{group}\;}{\mathrm{Diuretic}\;\mathrm{action}\;\mathrm{of}\;\mathrm{standard}\;\mathrm{group}}$$

#### 2.2.5. Stability Studies

The stability study of the ODTs containing the optimized FUR nanoparticles was performed using accelerated storage conditions (40 ± 2 °C and 75% ± 5% relative humidity) for 6 months according to the ICH guidelines, and an evaluation of the disintegration time, wetting time, and the IDR and DE_60_% values of the tablets after the 1st, 3rd, and 6th months of storage was carried out, and 6 tablets were used to evaluate the disintegration time, IDR, and DE_60_%, and 3 tablets were used to evaluate the wetting time.

#### 2.2.6. Statistical Analysis

One-way analysis of variance (ANOVA) was used for comparisons. A *p*-value < 0.05 was considered to indicate a significant difference.

## 3. Results and Discussion

### 3.1. Characterization of FUR Nanoparticles

#### 3.1.1. Effect of Different Stabilizer Types on FUR Nanoparticles’ Particle Size, Zeta Potential, and Dissolution Rate

Table 3 presents the effects of the different stabilizers (PVP K30, Povacoat, HPMC, Tween 80, PEG-6000, and pluronic F-127) on the particle size, zeta potential, and DE% of the FUR nanoparticles prepared by planetary ball milling. All the stabilized formulations were compared with non-stabilized formulations (FUR milled with no stabilizer). The particle size of the FUR nanoparticles prepared by planetary ball milling with water only and with no stabilizer used was 1170.33 nm (1.171 µm). This enlargement in size could be due to the high surface free energy, which can lead to particle aggregation [20]. The smallest particle size was observed with pluronic F-127, PVP K30, and HPMC, with sizes of 354.07 nm, 300.87 nm, and 470.73 nm, respectively. The PVP and HPMC stabilized the nanoparticles through steric stabilization, in which the polymers’ hydrophobic fraction was adsorbed onto the particle surface, thus forming a covering layer that prevented the particles from aggregating [21]. Pluronic F-127 is a block copolymer that is composed of hydrophilic and hydrophobic portions. The hydrophobic polypropylene oxide moieties could be adsorbed onto the particle surface, while the hydrophilic portion provided steric hindrance that prevented particle aggregation [22].

The stability of colloidal systems can be determined by the zeta potential. A low zeta potential was obtained with Tween 80 (−5.03 ± 3.82) (Table 3), and this was probably due to the formation of a thick adsorption layer, which covered and masked the particle charge [23]. On the other hand, the largest value was observed with PLU-F127(−25.3 ± 5.65), indicating a good system stability (Table 3).

**Figure 1:**
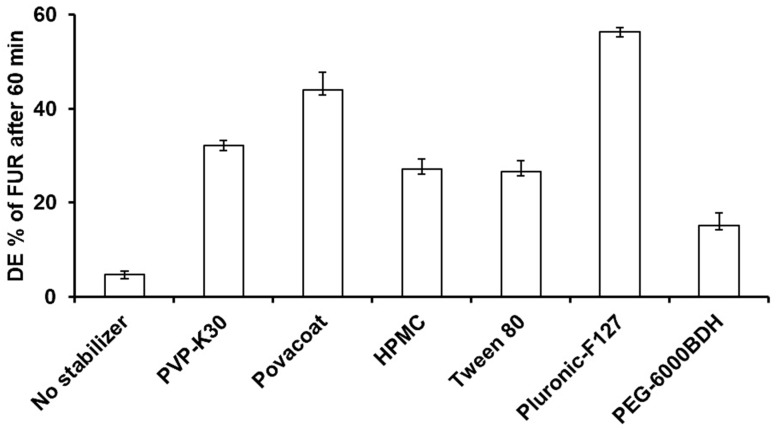
Influence of different stabilizer types on the dissolution efficiency of FUR nanoparticles prepared by planetary ball milling compared to non-stabilized FUR nanoparticles.

The dissolution efficiency of the FUR particles prepared by planetary ball milling without a stabilizer in phosphate buffer (pH 6.8) showed that less than 5% of the drug was dissolved within 1 h (Table 3 and Figure 1). In contrast, the results indicated that the percentage of the dissolution efficiency values of the stabilized nanoparticles were significantly higher than that of the non-stabilized microparticles, as they were 32.15%, 43.95%, 27.14%, 26.65%, and 56.34% for the PVP, Povacoat, HPMC, Tween 80, and pluronic F-127, respectively. The improvement in the drug dissolution rate for the nanoparticles stabilized by pluronic F-127 can be attributed to two effects. The co-effects of reducing the size of the particles by milling and the ability of the stabilizer to stabilize the system by preventing aggregation succeeded in improving the dissolution rate.

Based on the particle size, zeta potential, and the DE % results, PLU F-127 was selected as the best stabilizer, and the impact of its concentration was studied.

#### 3.1.2. Effect of Different Concentrations of Pluronic F-127 on the FUR Nanoparticles’ Particle Size, Zeta Potential, and Dissolution Rate

The effects of different pluronic F-127 concentrations (1%, 3%, and 5%) on the particle size, zeta potential, and DE% of the FUR nanoparticles are presented in Table 4. The data showed that the 3% PLU F-127 sample produced the smallest particle size of 354.07 ± 6.44 nm. Increasing the pluronic F-127 concentration level in the nanoparticles from 3% to 5% resulted in enlarging the particle size to 434.57 nm, which might have been due to increasing the viscosity of the solution, which impaired the ball’s movement, resulting in an insufficient milling process [24].

The data showed the same observation for the zeta potential, and increasing the PLU F-127 concentration from 3 to 5% decreased the zeta potential from −25.3 mV to −19.2 mV, respectively. The reason behind this could be the increase in the ionic strength of the suspension leading to intermolecular interactions [25].

**Figure 2:**
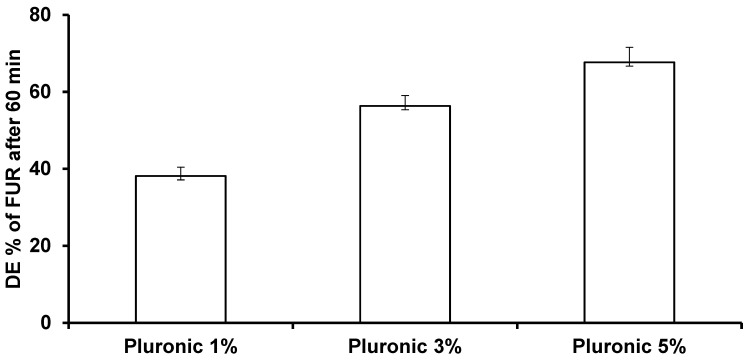
Effect of different concentrations of pluronic F-127 on the dissolution efficiency of FUR nanoparticles prepared by planetary ball milling.

Figure 2 and Table 4 illustrate the effect of utilizing different concentrations of PLU F-127 on the DE % of the FUR nanoparticles. It was clear that the dissolution rate and dissolution efficiency of the FUR from the nanoparticles increased with increasing the polymer concentration. This phenomenon might have been due to the solubilizing and wettability effect of the polymer. Pluronic F-127 is a non-ionic surfactant that can form micelles in the solution to improve the solubility of the drug [26]. For this reason, using a higher concentration of pluronic F-127 alone could improve the dissolution rate of the FUR nanoparticles because of its wettability properties.

Based on the previous results in terms of the particle size, zeta potential, and dissolution rate profile, it was found that a concentration of 3% showed the smallest particle size (354.07 ± 6.44), best zeta potential value (−25.3 ± 5.65), and percentage of dissolution rate (56.34%). Therefore, the FUR nanoparticle stabilized with 3% pluronic F-127 was selected as the best formulation and was subjected to physiochemical characterization studies.

### 3.2. Physicochemical Characterization of Optimized FUR Nanoparticles after Freeze-Drying

#### 3.2.1. Differential Scanning Calorimetry (DSC)

**Figure 3:**
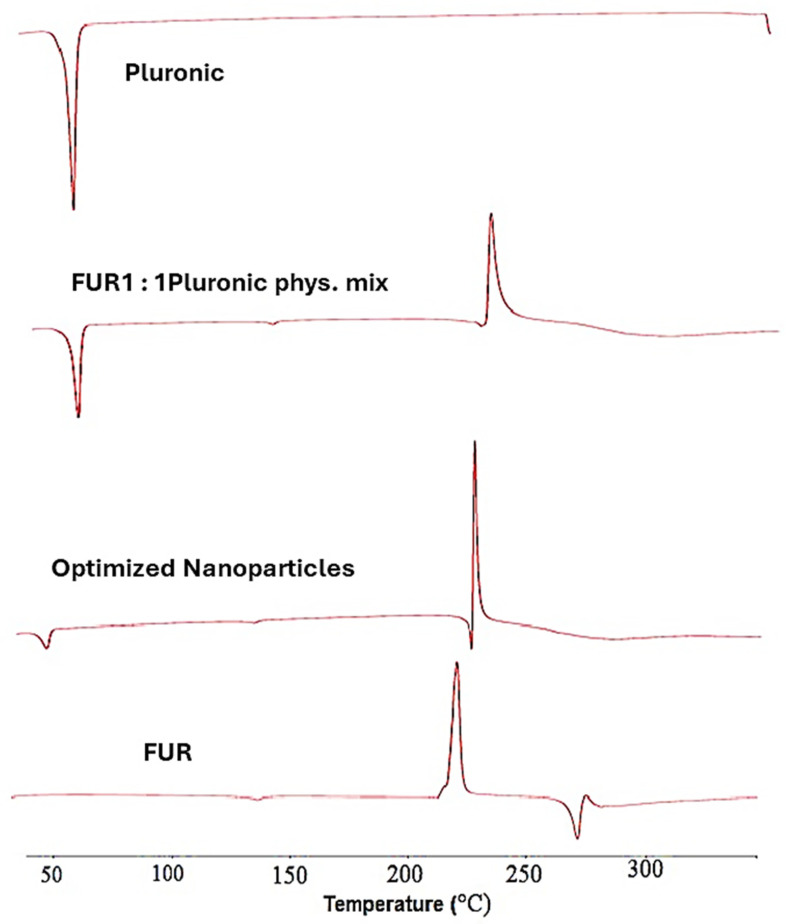
Differential scanning calorimetry scans of untreated FUR, pluronic F-127, FUR–pluronic F-127 physical mixture, and optimized FUR nanoparticle formula.

The thermograms in Figure 3 present the differential scanning calorimetry (DSC) scans of untreated FUR, pluronic F-127, a FUR–pluronic F-127 physical mixture, and the optimized FUR nanoparticle formula. The FUR showed an exothermic sharp peak at 220.34 °C, which was due to the drug decomposition [27]. Moreover, the endothermic peak that appeared at 271.40 °C was attributed to the melting of the FUR [28]. The pluronic F-127 exhibited a sharp melting endotherm at 59 °C.

The DSC scans of the FUR in its nanoparticle formulation revealed that the drug’s exothermic characteristic peak appeared at 227.57 °C. The peak itself was narrower than the one in the pure drug, and this could indicate a reduction in the crystallinity degree, and the links between the particles in the pure FUR decreased in the nanoparticle form, leading to a more amorphous shape. Similar results were obtained in the study by Ibrahim and El Sayeh. Furthermore, the DSC scans of the FUR in its selected nanoparticle formulation showed the complete disappearance of the endothermic characteristic peak of the drug compared to that of the untreated drug as well as the drug–polymer physical mixture. This can be explained by the solubility and homogeneous dispersion of FUR in the molten polymers [29].

#### 3.2.2. Fourier Transform Infrared Spectroscopy (FT)

**Figure 4:**
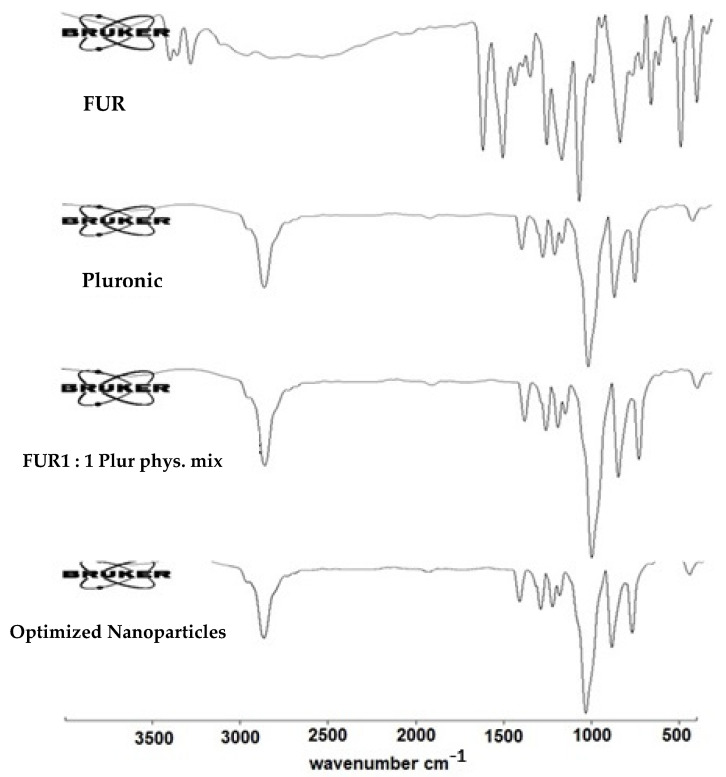
The Fourier transform infrared spectra of untreated FUR powder, pluronic F-127, FUR–pluronic F-127 1: 1 physical mixture, and optimized FUR nanoparticle formula.

Figure 4 shows the FT-IR spectra of the untreated FUR, pluronic F-127, FUR–pluronic F-127 physical mixture, and optimized FUR nanoparticle formula. The FTIR spectrum of the pure FUR presented vibrational bands with characteristic peaks around the wave number of 3600 cm^−1^ corresponding to carboxylic O-H stretching vibrations; it appeared as a very weak band, and the reason for this could be hydrogen bonding with the hydrogen in the NH group. In addition, two characteristic bands, one of which was forked and the other single, corresponding to secondary and primary NH groups were observed at 3389 cm^−1^ and 3277 cm^−1^, respectively [30]. Also, a characteristic band at around 1666 cm^−1^ was detected, which referred to the carboxylic C=O stretching vibration, and a band at 1173 cm^−1^ was observed, represented the stretching band corresponding to stretching C-O- bonds. In the case of the pluronic F-127, the FTIR spectrum of the polymer showed a characteristic large O-H sharp stretching band at 2875 cm^−1^ and a sharp band at 1099 cm^−1^ related to C-O stretching vibrations. Moreover, the FTIR for the optimized FUR nanoparticle formula showed that the intensity of the OH band of the pluronic F-127 was not significantly changed. In addition, no other changes could be detected for both the FUR and the polymer FTIR bands, indicating the absence of FUR–pluronic F-127 interactions.

#### 3.2.3. X-ray Diffraction Analysis

**Figure 5:**
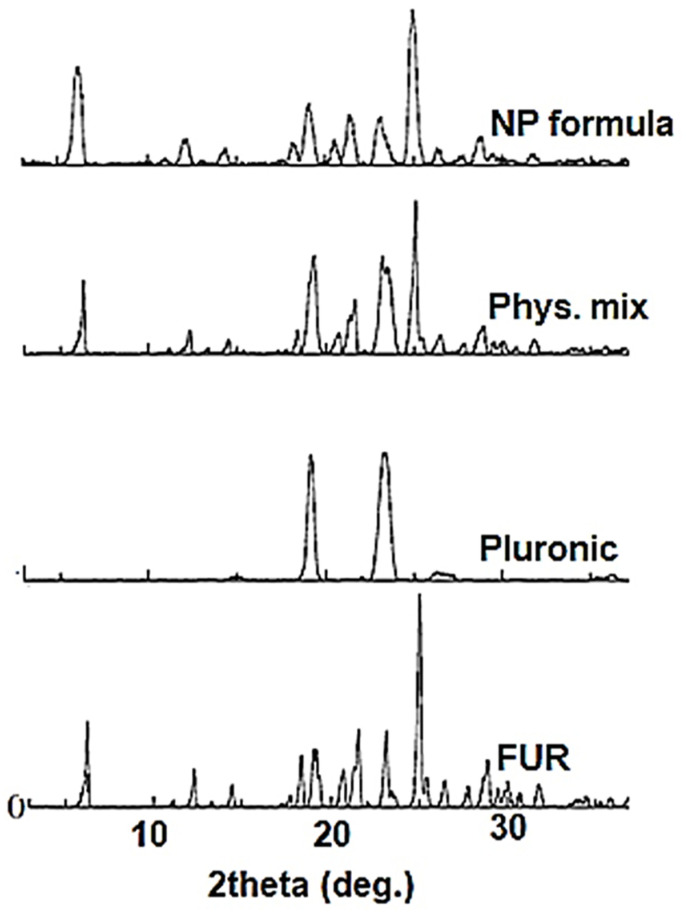
X-ray powder diffraction spectra of untreated FUR powder, pluronic F-127, FUR–pluronic F-127 physical mixture, and optimized FUR nanoparticle formula.

X-ray powder diffraction spectra of the pure FUR, pluronic F-127, FUR–pluronic F-127 physical mixture, and optimized FUR nanoparticle formula were obtained and are presented in Figure 5. The diffractogram of the FUR showed sharp peaks at diffraction angles (2Ɵ°) of 6.24, 19.18, 21.58, 23.14, 25.02, and 28.88 degrees, with diffraction intensities of 883, 595, 805, 789, and 2187, respectively [31], which was agreeing with the reported powder diffraction data. The pluronic F-127 showed only two intense diffraction peaks at 2Ɵ° of 19.18 and 23.38, with diffraction intensities of 2547 and 2639, respectively. The XRPD spectra of the FUR nanoparticles and the FUR–pluronic F-127 physical mixture showed that no changes in the drug crystallinity could be observed, as the drug diffraction peaks in the two samples were the summation of the diffraction peaks of the drug and the nanoparticle-stabilizing polymer.

### 3.3. ODT Formulations Loaded with FUR Nanoparticles by the Sublimation Method

A full factorial design (3^2^) was utilized to study the impact of different concentrations of thymol (sublimating agent) and CPV (superdisintegrant) on the properties of different matrices. Table 5 presents the properties of the different formulations.

#### 3.3.1. Effect of Each Independent Parameter on the Disintegration Time

Table 6 shows the statistical analysis utilizing ANOVA. The independent factors, thymol (A) and CPV (B), both showed insignificant effects (*p* = 0.865 and 0.42, respectively) on the disintegration time of the ODT formulations. Generally, the figure shows that increasing the CPV concentration increased disintegration time as well, as shown in the case of F4 and F5, where the disintegration time increased from 8 s to 11 s, respectively. Formulations F2 and F3, in which the CPV concentration increased from 2 to 6%, showed that increasing the CPV concentration to a certain limit decreased the disintegration time from 10 to 7 s, respectively.

**Figure 6:**
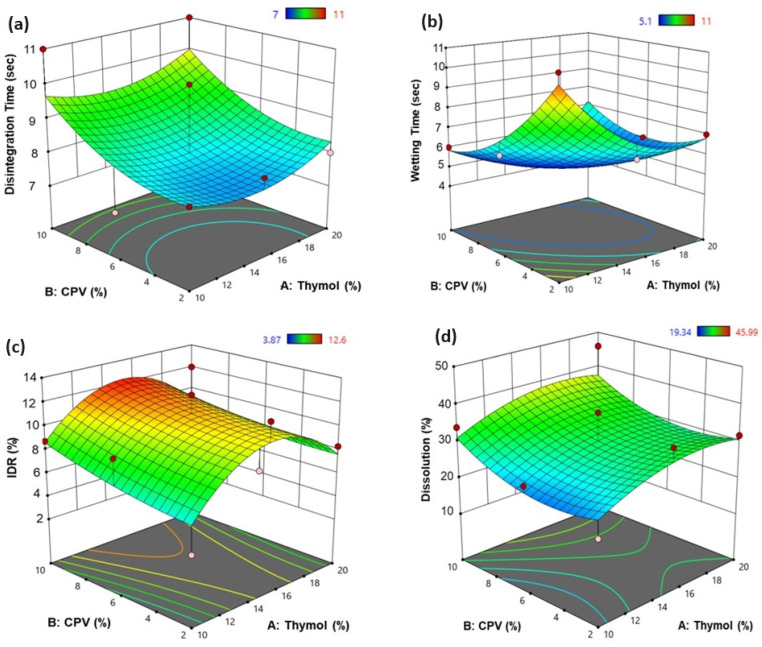
Three-dimensional response surface plot for the effect of independent variables thymol and CPV on the response parameters, (**a**) disintegration time; (**b**) wetting time; (**c**) IDR %, and (**d**) dissolution %.

Figure 6a presents the 3D response surface plot, estimating the effect of thymol and CPV on the disintegration time of ODT formulations loaded with FUR NPs. It shows that the thymol and CPV exhibited an insignificant agonistic effect on the drug disintegration time, in which a prolonged disintegration time was reported when using the highest level of thymol and the highest level of CPV, as observed in F5 using a 20% concentration of thymol and a 10% concentration of CPV, whose disintegration time was equal to 11 s. The shortest disintegration time (7 s) was recorded in the case of F6 using a 10% concentration of thymol and a 6% concentration of CPV.

#### 3.3.2. Effect of Each Independent Parameter on the Wetting Time

The analysis of variance of the effect of the independent parameters on the wetting time of the ODT formulations loaded with FUR NPs (Table 6) showed a significant effect of CPV and an interactive effect (*p* = 0.0414, 0.0462, respectively) on the wetting time of the ODT formulations.

The response surface plot (Figure 6b) showed that the highest wetting time value (11 s) was recorded in the case of ODT formula F8, in which the lowest level of both CPV, 2%, and thymol, 10%, were applied. In contrast, the highest level of both thymol (A) and CPV (B), i.e., 20% and 10% respectively, resulted in a decreased wetting time (7 s), as observed in the case of ODT formula F5. The reason could be regarding the pores that formed in the tablet after the sublimation process, which allowed for more water to penetrate and get absorbed, which will led to a short wetting and dissociation time [32].

#### 3.3.3. Effect of Each Independent Parameter on the Initial Dissolution Rate

The ANOVA analysis showed that the individual effects of thymol and CPV as well as their interactive and quadratic effects were insignificant (Table 6).

Figure 6c shows the 3D response plot, and it illustrates that the highest IDR (12.6 ± 4.66% after 5 min) of FUR from the ODT formulations was noticed in the case of ODT formula F2, which was prepared by using a 15% concentration of thymol along with a 6% concentration of CPV. In contrast, the lowest IDR value (3.87 ± 0.11%) of FUR from the ODT formulations was recorded with ODT formulation F8, which was prepared by using the lowest level of both CPV (B), 2%, and thymol (A), 10%. The presence of superdisintegrants in the tablet formulations enhanced the initial dissolution rate due to the high permeability of the tablet that could be obtained [33,34].

#### 3.3.4. Effect of Each Independent Parameter on the Dissolution Efficiency, DE_60_%

The ANOVA analysis showed that the individual effects of thymol and CPV as well as their interactive and quadratic effects were insignificant (Table 6). Table 5 shows that the DE% increased from 19.34 ± 2.23% to 34.88 ± 0.16% with increasing the thymol concentration from 10 (F8) to 15% (F1), respectively. Increasing the thymol concentration from 15% (F1) to 20% (F4) reduced the DE% to 31.73 ± 0.65%.

The 3D response plot (Figure 6d) shows that the thymol and CPV exhibited an agonistic effect on the drug dissolution, in which the highest value (45.99 ± 4.5%) was reported when using the highest level of thymol and the highest level of CPV, as observed in F5 using a 20% concentration of thymol and a 10% concentration of CPV. The lowest value of dissolution (19.34 ± 2.23%) was recorded in the case of F8 using a 10% concentration of thymol and where the lowest level of CPV was 2%. Apparently, due to the sublimation, the thymol left the tablet matrix, thus forming a void or pores that increased the tablet porosity, through which the water could enter, leading to fast disintegration. The addition of the superdisintegrant may have also played a crucial role in enhancing the dissolution rate. Mahrous et al. showed that the presence of superdisintegrants may enhance water permeation (wicking action) into tablets, which prompts the wetting action, shortens the disintegration time, and finally causes a fast dissolution rate [35].

**Figure 7:**
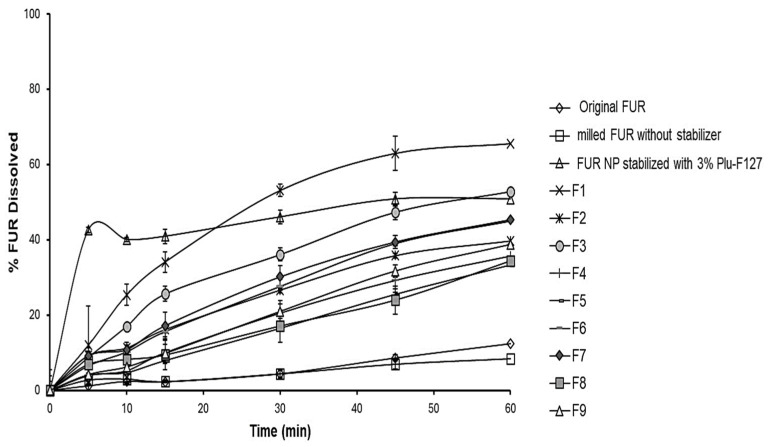
Dissolution profiles of different ODT formulations loaded with FUR NPs.

From the cumulative % dissolved vs. time plot (Figure 7), it was found that all the ODT formulations showed better results and higher values of the dissolution profile than the original FUR and the non-stabilized one. The co-effects of reducing the size of the particles by milling and the ability of the stabilizer to stabilize the system by preventing aggregation and to form rapidly disintegrating tablets succeeded in improving the dissolution rate.

### 3.4. Optimization of FUR ODT

The optimization procedure was carried out based on a certain desirability of the response parameters, which were a short disintegration time, a short wetting time, and a maximum value of IDR and DE%. Based on the modeling carried out by Stat-Ease, version 11, the optimal formulation was composed of thymol (A) = 15.39% and CPV (B) = 9.13%.

Table 7 shows the comparison between the predicted and observed values of the evaluated optimized formulation.

The observed values were found to be close to the predicted optimized values of the ODT formula. The observed disintegration time was 10 s, the observed wetting time was 5.7 s, the IDR was 10.29 ± 2.15, and the observed %DE_60_ was 22.14 ± 2.64%.

### 3.5. Pharmacodynamics Evaluation of Diuretic Activity

The volume of urine excreted upon oral administration of FUR from albino Wistar rats at different time intervals was collected and calculated to examine its diuretic activity profiles.

**Figure 8:**
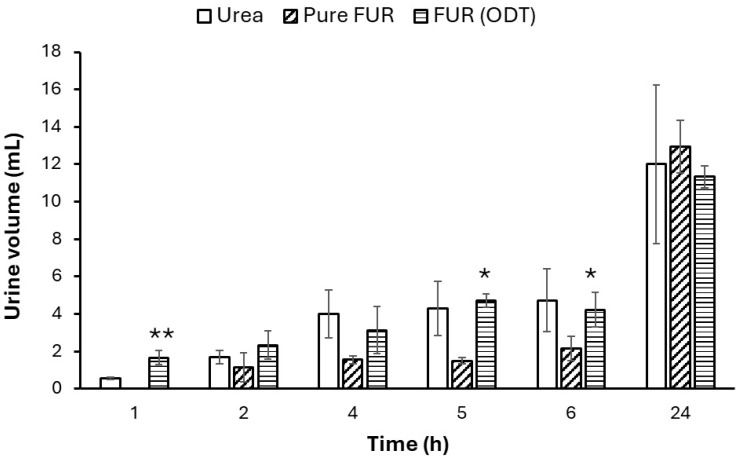
Volume of urine excreted volume vs. time histogram of FUR after oral administration of its optimized ODT formulations loaded with FUR NPs in comparison to the pure drug and Urea, * considered significant compared with pure FUR. (* *p* < 0.05, ** *p* < 0.01).

It was observed that the urine output of the urea (control) group was high compared to the second group, to which untreated FUR was administered for 6 h, as shown Figure 8; in contrast to the amount after 24 h, the untreated FUR group increased the amount to more than the urea group, and this explains the slow onset of FUR [36]. It is important to mention that the urination started after the second hour after the FUR administration and was kept constant for 5 h, after which it then started to increase to reach 12.95 ± 0.07 mL after 24 h compared with 12 ± 4.24 mL with the urea group. The % of urine excretion after 24 h was 7.02 ± 0.933% with the FUR group compared to 7.28 ± 0.622% with the urea group, which was insignificant (*p* = 0.32). The diuretic action of FUR compared to urea was 96.15%.

On the other hand, comparing the urea (control) group with the one administered with ODTs loaded with FUR NPs, it was clear that the urination activity was significantly higher in the ODTs group than in the urea group in the first hours, as it was 1.66 mL and 0.55 mL, respectively. In the second hour, the urinary excretion volume was 2.33 mL for the group treated with ODTs loaded with FUR NPs and 1.7 mL for the urea group. The urinary excretion volume reached 11.33 ± 0.57 mL after 24 h of administering the ODT formulation, as shown in Figure 8. The % of urine excretion after 24 h was 10.82 ± 1.04% for the ODT formulation compared to 7.28 ± 0.622 % for urea (*p* = 0.033) and 7.02 ± 0.933% for FUR (*p* = 0.059). The results showed that the diuresis (expressed as the urine volume) was higher in the animal group treated with ODTs loaded with FUR NPs for more than 6 h, showing a potentially better diuretic effect compared to the untreated FUR group, as shown in Figure 8, and the reason behind this is that the reduction in the particle size (nanoparticles) increased the solubility and thus enhanced the absorption process as well as causing the fast dissolution and absorption of the drug, offering a rapid onset of action of the ODTs [37].

**Figure 9:**
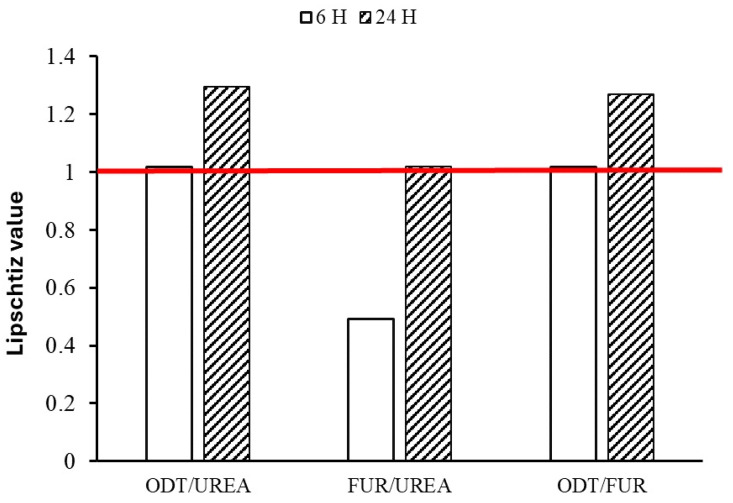
Lipschitz’s value (test/control).

Figure 9 shows the time response plot based on Lipschitz’s theory. The results are expressed as the ‘Lipschitz value’, that is, the ratio T/U, in which T is the response of the test compound and U is that of the urea treatment. A value of 1.0 or more is regarded as a positive effect. The results showed that the Lipschitz value for ODT/urea was 1 after 6 h and reached 1.2 after 24 h. By comparing the ODT with FUR, it was found that after 24 h, the Lipschitz value was 1.26, while FUR/urea showed the inefficiency of FUR after 6 h (0.49). The value almost reached 1 after 24 h.

Table 8 presents the diuretic activity of the different groups in terms of diuretic excretion %, diuretic action, and diuretic index after 6 and 24 h. The diuretic action of the ODT formulation after 6 h of administration was 1.01 compared to 0.31 for FUR. The diuretic action increased by 0.72-fold after the administration of the ODT formulation loaded with FUR NPs. After 24 h, the diuretic action of the FUR NPs in the ODT formulation increased by 0.74-fold after the pure FUR reached its maximum of 0.90 compared to 1.57 for the ODT formulation. The diuretic index was 1.73 for the ODT over pure FUR.

The pharmacodynamics study showed promising results, with a superiority of ODT over pure FUR. The diuretic action of the pure FUR started just after 24 h, while the diuretic action of the ODT loaded with FUR NPs started after 1 h after the administration. We can conclude that the increment in the diuretic activity after administering the ODT formulation could be due to several factors. First the ODT promoted the disintegration of the drug, resulting in a rapid onset of action. The second reason, which may have had a pronounced effect, was the formulation of the FUR as nanoparticles. The nanoparticles may have enhanced the solubility and the dissolution rate, which was reflected in the pronounced diuretic effect.

## 4. Conclusions

This work studied the impact of different stabilizer biomaterials on furosemide nanoparticles prepared by planetary ball milling, and it was found that pluronic F-127 at 3% *w*/*v* was the optimum stabilizer in terms of the particle size, zeta potential, and dissolution efficiency. In addition, reducing the size of the furosemide drug particles to nano-size and incorporating it into orally disintegrating tablets led to an actual increase in the dissolution rate as well as in the bioavailability of the drug in the body. The preparation of orally disintegrating tablets with nanoparticles of furosemide improved the in vitro dissolution rate of the drug and, in turn, enhanced its pharmacological response. The laboratory analysis results showed that diuresis was higher in the group of rats treated with the orally disintegrating tablets loaded with furosemide nanoparticles for more than 6 h, indicating a better diuretic effect compared to the furosemide-untreated group.

## Figures and Tables

**Table 1:** The independent factors for the formulation of ODTs by sublimation.

Independent Parameters	Low Level (−1)	Medium Level (0)	High Level (+1)
Thymol (A) (%)	10	15	20
CPV (B) (%)	2	6	10
Dependent Parameters Disintegration time (S) Wetting time (S) IDR (%) DE (%)			

**Table 2:** Composition of 200 mg ODT containing FUR nanoparticles prepared by sublimation method.

Formula	F1	F2	F3	F4	F5	F6	F7	F8	F9
FUR NPs	Equivalent to 20 mg								
Thymol (%)	15	15	15	20	20	10	10	10	20
CPV (%)	2	6	10	2	10	6	10	2	6
Sodium Stearyl Fumarate (SSF)	1%								
Filler (Avicel–Lactose) (1:1)	To 200 mg								

**Table 3:** Screening data of the effect of different stabilizers on attributes of furosemide nanoparticles prepared by planetary ball milling by using 500 rpm as milling speed, milling ball size of 0.5 mm, and three milling cycles (each of 10 min with 5 min pause).

Stabilizer	Type of Stabilizer	Particle Size Avg ± SD (nm)	Zeta Potential (mV)	DE_60_%
**No stabilizer**	-	1170.33 ± 30.99	−6.72 ± 4.42	4.77 ± 0.71
**PVP K30**	Steric	300.87 ± 9.13	−10.3 ± 5.81	32.15 ± 1.13
**Povacoat**	Steric	559.17 ± 19.93	−14.9 ± 4.07	43.95 ± 3.83
**HPMC**	Steric	470.73 ± 76.38	−11.5 ± 4.11	27.14 ± 2.12
**Tween 80**	Static	655.53 ± 46.92	−5.03 ± 3.82	26.65 ± 2.20
**PEG-6000 BDH**	Steric	2053 ± 264.78	−32.6 ± 4.8	15.17 ± 0.95
**Pluronic F-127**	Steric	354.07 ± 6.44	−25.3 ± 5.65	56.34 ± 2.68

**Table 4:** Screening data of the effect of different concentrations of pluronic F-127 on attributes of FUR nanoparticles prepared by planetary ball milling by using 500 rpm as milling speed, milling ball size of 0.5 mm, and three milling cycles (each of 10 min with 5 min pause).

Stabilizer	Particle Size (nm)	Zeta Potential (mV)	DE_60_%
No stabilizer	1170.33 ± 30.99	−6.72 ± 4.42	4.77 ± 0.71
Pluronic F127 1%	412.73 ± 45.14	−21.3 ± 4.42	38.13 ± 2.32
Pluronic F-127 3%	354.07 ± 6.44	−25.3 ± 5.65	56.34 ± 2.68
Pluronic F-127 5%	434.57 ± 40.17	−19.2 ± 6.23	67.67 ± 3.89

**Table 5:** Properties of ODT formulations loaded with FUR nanoparticles.

Formula (Thymol/CPV) (%)	Drug Content (AV)	Weight Variation	Disintegration Time (s)	Wetting Time (s)	IDR%	DE_60_%
F1 (15/2)	5.5	0.1912	8	6.5	12.16 ± 2.01	34.88 ± 0.16
F2 (15/6)	10.99	0.1784	10	5.8	12.6 ± 4.66	37.87 ± 2.59
F3 (15/10)	7.07	0.18793	7	5.1	9.17 ± 1.03	26.56 ± 1.66
F4 (20/2)	13.31	0.1922	8	7	8.31 ± 1.09	31.73 ± 0.65
F5 (20/10)	12.6	0.1989	11	7	12.01 ± 5.4	45.99 ± 4.5
F6 (10/6)	14.27	0.1948	7	6.5	9.24 ± 2.3	24.98 ± 0.64
F7 (10/10)	3.17	0.1978	11	6	8.74 ± 0.83	33.88 ± 1.99
F8 (10/2)	15	0.1901	8	11	3.87 ± 0.11	19.34 ± 2.23
F9 (20/6)	13.42	0.2039	8	6	4.17 ± 1.00	20.51 ± 1.62

**Table 6:** Analysis of variance of the effect of independent parameters on dependent parameters of ODT formulations loaded with FUR NPs.

	Source	Mean Square	F-Value	*p*-Value
Disintegration Time	A-Thymol	0.166	0.03	0.8653
	B-CPV	4.17	0.85	0.4240
	AB	0.00	0.00	1.0000
	A^2^	0.50	0.10	0.7701
	B^2^	0.50	0.10	0.7701
Wetting Time	A-Thymol	2.04	3.53	0.1570
	B-CPV	6.83	11.79	0.0414
	AB	6.25	10.80	0.0462
	A^2^	4.21	7.26	0.0741
	B^2^	2.00	3.45	0.1600
IDR %	A-Thymol	1.16	0.0704	0.8080
	B-CPV	5.19	0.3144	0.6142
	AB	0.342	0.0207	0.8946
	A^2^	25.73	1.56	0.3004
	B^2^	0.278	0.0169	0.9048
DE %	A-Thymol	66.87	0.5068	0.5279
	B-CPV	69.91	0.5298	0.5193
	AB	0.0196	0.0001	0.9910
	A^2^	27.36	0.2073	0.6798
	B^2^	36.58	0.2772	0.6350

**Table 7:** Composition of the optimized FUR ODT formula, the desirability of responses, and their observed and predicted values.

Optimized Formula Composition	Response			
	Type	Desirability	Predicted	Observed
Thymol (A) = 15.39% CPV (B) = 9.13%	Y1: Disintegration time (s)	Minimum	8.97	10
	Y2: Wetting time (s)	Minimum	4.95	5.7
	Y3: IDR (%)	Maximum	12.013	10.29 ± 2.15
	Y4: DE_60_ (%)	Maximum	35.78	22.14 ± 2.64

**Table 8:** Diuretic activity of ODT formulations loaded with FUR NPs compared to pure FUR (standard) and urea (control) after 6 and 24 h.

Group	Urinary Excretion (%)		Diuretic Action		Diuretic Index	
	6th Hour	24th Hour	6th Hour	24th Hour	6th Hour	24th Hour
Group I, urea	7 ± 0.22	7.28 ± 0.62	NA	NA	NA	NA
Group II, pure FUR	2.18 ± 0.91	6.62 ± 0.36	0.31	0.90	NA	NA
Group III, ODT FUR NPs	7.08 ± 0.11	11.48 ± 0.11	1.01	1.57	3.23	1.73

NA: Not Applicable.

## Data Availability

All the data are available in the manuscript.
